# Scale of differentiated service delivery implementation in HIV care facilities in low‐ and middle‐income countries: a global facility survey

**DOI:** 10.1002/jia2.26477

**Published:** 2025-07-07

**Authors:** Nathalie Verónica Fernández Villalobos, Fabrice Helfenstein, Vohith Khol, Christella Twizere, Mayara Secco, Barbara Castelnuovo, Jacqueline Huwa, Thierry Tiendredbeogo, C. William Wester, Siew Moy Fong, Gad Murenzi, Yanink Caro‐Vega, Rita Elias Lyamuya, Idiovinio Rafael, Djimon Marcel Zannou, Kathy Petoumenos, Dominique Mahambou Nsonde, Jorge Pinto, Kara Wools‐Kaloustian, Carolyn Bolton Moore, Ounoo Elom Takassi, Sasisopin Kiertiburanakul, Rogers Ajeh Awoh, Shamim M. Ali, Geoffrey Fatti, Karen Malateste, Elizabeth Zaniewski, Marie Ballif, the International epidemiology Databases to Evaluate AIDS

**Affiliations:** ^1^ Institute of Social and Preventive Medicine University of Bern Bern Switzerland; ^2^ Department of Clinical Research University of Bern Bern Switzerland; ^3^ National Center for HIV/AIDS, Dermatology & STDs Phnom Penh Cambodia; ^4^ Centre National de Référence en matière de VIH/SIDA (CNR) Bujumbura Burundi; ^5^ Instituto Nacional de Infectologia Evandro Chagas (INI) Fundação Oswaldo Cruz Rio de Janeiro Brazil; ^6^ Infectious Diseases Institute, College of Health Sciences Makerere University Kampala Uganda; ^7^ Lighthouse Trust Lilongwe Malawi; ^8^ University of Bordeaux National Institute for Health and Medical Research (INSERM) UMR 1219 Research Institute for Sustainable Development (IRD) EMR 271 Bordeaux Population Health Research Centre Bordeaux France; ^9^ Division of Infectious Diseases Department of Medicine Vanderbilt University Medical Center (VUMC) Nashville Tennessee USA; ^10^ Department of Pediatrics Hospital Likas Kota Kinabalu Malaysia; ^11^ Einstein‐Rwanda Research and Capacity Building Program, Research for Development and Rwanda Military Referral and Teaching Hospital Kigali Rwanda; ^12^ Departamento de Infectología Instituto Nacional de Ciencias Médicas y Nutrición Salvador Zubirán Ciudad de México México; ^13^ Morogoro Regional Hospital – CTC Indiana University Morogoro Tanzania; ^14^ SolidarMed Pemba Mozambique; ^15^ Centre national de référence pour la recherche et la prise en charge des PVVIH au Centre National Hospitalier Universitaire HK MAGA (CNHU‐HKM) Cotonou Bénin; ^16^ The Kirby Institute, UNSW Sydney Sydney New South Wales Australia; ^17^ Centre de Traitement Ambulatoire of Brazzaville Brazzaville Congo; ^18^ Universidade Federal de Minas Gerais (UFMG) Belo Horizonte Brazil; ^19^ Department of Medicine Indiana University School of Medicine Indianapolis Indiana USA; ^20^ Centre for Infectious Disease Research in Zambia (CIDRZ) Lusaka Zambia; ^21^ University of Alabama at Birmingham Birmingham Alabama USA; ^22^ Lome University Lome Togo; ^23^ Faculty of Medicine Ramathibodi Hospital Mahidol University Bangkok Thailand; ^24^ University of Buea Buea Cameroon; ^25^ School of Medicine, College of Health Sciences Moi University Eldoret Kenya; ^26^ Kheth'Impilo AIDS Free Living Cape Town South Africa; ^27^ Division of Epidemiology and Biostatistics, Department of Global Health Faculty of Medicine and Health Sciences Stellenbosch University Cape Town South Africa; ^28^ Department of Infectious Diseases Bern University Hospital and University of Bern Bern Switzerland

**Keywords:** antiretroviral therapy, differentiated service delivery, HIV, key and vulnerable populations, low‐ and middle‐income countries, patient‐centred care

## Abstract

**Introduction:**

In 2016, the World Health Organization recommended differentiated service delivery (DSD) as a client‐centred approach to simplify HIV care in frequency and intensity, thus reducing the clinic visit burden on individuals and HIV programmes. We describe the scale of DSD implementation among HIV facilities in low‐ and middle‐income countries (LMICs) in Latin America, Africa and the Asia‐Pacific before the COVID‐19 pandemic.

**Methods:**

We analysed facility‐level survey data from HIV care facilities participating in the International epidemiology Databases to Evaluate AIDS consortium in 2019. We used descriptive statistics to summarise the availability of DSD, multi‐month dispensing (MMD) and DSD for HIV treatment models. We explored factors associated with DSD implementation using multivariable models.

**Results:**

We included 175 facilities in the Asia‐Pacific (*n* = 30), Latin America (*n* = 8), Central Africa (*n* = 21), East Africa (*n* = 74), Southern Africa (*n* = 28) and West Africa (*n* = 14). Overall, 133 facilities (76%) reported implementing DSD. Of these, 91% offered DSD for HIV treatment, 61% for HIV testing and 59% for antiretroviral therapy (ART) initiation. The most common duration of ART refills for clinically stable clients was 3MMD, (70%), followed by monthly (14%) and 6MMD (10%). Facility‐based individual models were the most frequently available DSD for the HIV treatment model (82%), followed by client‐managed group models (60%). Out‐of‐facility individual models were available at 48% of facilities. Facility‐based individual models were particularly common among facilities in East (92%) and Southern Africa (96%). Facilities in medium and high HIV prevalence countries, and those with 3MMD, were more likely to implement DSD.

**Conclusions:**

In 2019, DSD was available in most HIV care facilities globally but was not evenly implemented across regions and HIV services. Most offered facility‐based DSD for HIV treatment models and 3MMD for clinically stable clients. Efforts to expand DSD for HIV testing and ART initiation and to offer longer MMD can improve long‐term retention in care of people living with HIV in LMICs, while further alleviating the operational burden on healthcare services. These findings from the pre‐COVID‐19 era underline the need for strengthening DSD in HIV care, which remains at the centre of current efforts towards client‐centred care.

## 1 INTRODUCTION

Recommended by the World Health Organization (WHO) since 2016 [1], differentiated service delivery (DSD) is a healthcare approach that aims to provide greater flexibility and capacity to better manage an increasingly large and diverse population of people living with HIV (PWH) [1]. In contrast to a “*one size fits all*” approach [2], DSD is a client‐centred approach that strives to simplify and adjust HIV services across the cascade of HIV care based on the preferences and needs of individuals seeking care [3, 4, 5]. DSD can be applied across the entire HIV care cascade by offering targeted interventions such as pre‐exposure prophylaxis, simplifying treatment initiation with differentiated follow‐up schedules, improving retention by offering multi‐month dispensing (MMD) and flexible clinical consultation systems, and facilitating re‐engagement in care through decentralised service delivery points, task shifting and peer‐led support [3, 6]. DSD for HIV treatment can be defined within one of the following models: group models managed by healthcare workers, group models managed by clients, individual models based at facilities and individual models not based at facilities [6]. Each model aims to address the various clients’ concerns. Facility‐based models with MMD and fast‐track services may minimise the risk of sharing an individual's HIV status to others besides healthcare workers and minimise waiting times [7, 8]. In contrast, community‐based models may reduce travel costs and waiting times for PWH and have the additional benefit of lowering staff workload and congestion at health facilities [9, 10].

Coinciding with the DSD recommendations in 2016, the WHO began advocating for less frequent clinical consultations and extended MMD for clinically stable clients who attained viral suppression. Extended MMD contributes to reducing the time and financial burden of care for clients [11, 12] and to reducing clinic congestion. It also allows the reallocation of resources to other specialised services targeted towards populations with distinct needs [11, 13], including clients with advanced disease [1, 5, 14]. The implementation of national policies on DSD can be influenced by different factors such as the capacity and funding of HIV programmes, the availability and training of healthcare workers and the decentralisation of services to the community. These factors collectively can impact the effectiveness of DSD policy implementation in various settings [9].

While previous studies have explored the regional implementation of DSD in HIV care [12, 15, 16, 17, 18, 19, 20], little is known about the global rollout of DSD in resource‐constrained settings, which carry most of the HIV burden. In this study, we described the implementation of DSD before the effects of COVID‐19 in a large sample of HIV treatment and care facilities in low‐ and middle‐income countries (LMICs) across Latin America, Africa and Asia‐Pacific. We summarised the availability of DSD for HIV treatment models and explored factors associated with the probability of implementing DSD. Additionally, we examined the frequency of MMD and the availability of dedicated HIV clinics for different population groups. Our analysis provides a global overview of DSD implementation in the pre‐COVID‐19 era.

## 2 METHODS

### 2.1 Study design

We conducted a cross‐sectional study within the International epidemiology Databases to Evaluate AIDS (IeDEA) consortium. IeDEA is an international research collaboration that collects data from HIV care and treatment programmes in 44 countries in seven geographic regions: the Asia‐Pacific; the Caribbean, Central and South America (Latin America); Central, East, Southern and West Africa; and North America [21]. As described elsewhere [22], IeDEA comprises a heterogenous combination of HIV care and treatment facilities at academic and community‐based hospitals and health centres, including facilities that provide specialised HIV care and serve a large number of clients, as well as numerous facilities in Southern Africa that are part of large HIV treatment programmes.

We used data from a site‐level survey that was conducted from September 2020 to March 2021 on a global sample of 238 HIV treatment and care facilities that participated in the IeDEA consortium in 2019. The survey explored standard practices and services in facilities prior to the COVID‐19 pandemic (Supporting information Text S2). It used standardised online questionnaires that were available in English and French [22, 23]. All facilities that were contributing data to IeDEA in 2020 were eligible for the survey. In IeDEA's Southern Africa region, where large HIV programmes contribute data for numerous facilities, approximately 15% of participating facilities were included based on a combined convenience and stratified random sample of all facilities in the region [22]. The convenience sample included facilities that had responded to prior IeDEA‐wide surveys to allow for longitudinal analyses of HIV care and service attributes across time, whereas the stratified random sample was drawn from facilities that are part of large programmatic cohorts, with the number of urban versus rural sites reflecting each cohort's distribution of facilities.

The survey included questions to assess the type and availability of differentiated services (HIV testing, antiretroviral therapy [ART] initiation and HIV treatment); the characteristics of clients eligible for DSD for HIV treatment (e.g. returning clients, clients stable on ART, clients with virologic failure); and the type of DSD for HIV treatment models available at the facility (e.g. patient‐managed groups, healthcare worker managed groups, facility‐based individual models, out‐of‐facility‐based individual models). The survey questionnaire was distributed via paper forms and via a REDCap survey – a web‐based software platform for secure data capture in research studies [24, 25]. For facilities that completed the survey using the paper forms, responses were entered into the REDCap electronic database by local or regional data managers.

### 2.2 Eligibility criteria

We included survey results from facilities located in LMICs, as defined by the World Bank in 2019 [26]. We excluded facilities that did not complete the survey, meaning no information was provided by these facilities.

### 2.3 Definitions

We obtained national HIV prevalence from the UNAIDS 2019 estimates for adults aged 15−49 [27] for all countries except Mozambique and China, where data was unavailable. For Mozambique, we obtained HIV prevalence estimates from the CDC Country Profile [28], and for China from the Global Burden of Diseases (GBD) study [29]. We categorised HIV prevalence as low (< 1%), medium (1−4.9%) or high (≥ 5%). We categorised facilities based on the level of care provided, and facility type was defined as either health centre; district hospital; regional, provincial or university hospital; or unknown. We defined the residence of the population served by facilities as predominantly urban, mixed urban/rural or predominantly rural. This definition was based on facilities’ own assessment of how they would describe the residence of the population served. We categorised age groups served at the facility as children (0−9 years of age), adolescents (10−24 years of age) and adults (≥ 20 years of age). Provision of DSD and type of DSD provided including HIV testing, ART initiation and HIV treatment were categorised as binary variables (yes/no). Standard frequency of refills/MMD was defined as monthly, every 3 months, every 6 months or other. We categorised DSD for HIV treatment models as: client‐managed groups (e.g. community ART refill group, community patient‐led ART delivery, community adherence group, peer support group), healthcare worker‐managed groups (e.g. ART adherence clubs, patient adherence club, youth club, teen club), facility‐based individual models (e.g. fast track, quick pick‐up, pharmacy refill without clinical consultation) and out‐of‐facility individual models (e.g. mobile outreach, fixed community ART distribution points, community pharmacy, home delivery). We defined key population groups as gay men and other men who have sex with men, female sex workers, transgender individuals, people who inject drugs and incarcerated people or in other closed settings such as in detention centres or institutional care facilities.

### 2.4 Statistical methods

We used descriptive statistics to summarise the characteristics of facilities, stratified by region. We ran univariable models with the implementation of DSD (no/yes) as the dependent variable and each of the following factors as the independent variable: country income designation, HIV prevalence, facility type, residence of the population served and age group of clients served (children [yes/no], adolescents [yes/no] and adults [yes/no]). We assumed binomial distribution of the error and used a logit link function. We considered the country where the facilities were located (*n* = 35) as a random factor to account for the non‐independence of facilities within the same country. We built a multivariable generalised linear mixed‐effect model with the implementation of DSD (no/yes) as the dependent variable, all factors listed above as independent variables and country as the random factor. For this multivariable model, we computed marginal odds ratios that allow a comparison of the association between a given explanatory factor and the implementation of DSD across different studies and data sets [30].

All statistical analyses were performed using RStudio version 4.2.2 [31], and the geographical representation was done using ArcGIS version 10.8.2 using the boundaries provided by ESRI [32]. The graphical representation of the analysis was performed using the packages ggplot2 [33] and gridExtra [34].

### 2.5 Ethics

The site‐level survey was designated as a non‐human subjects operational/quality improvement project by the Vanderbilt University Medical Center (VUMC) Institutional Review Board (#200013) [22].

## 3 RESULTS

**Figure 1:**
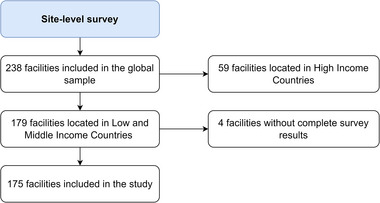
Flow diagram of included HIV care facilities.

**Figure 2:**
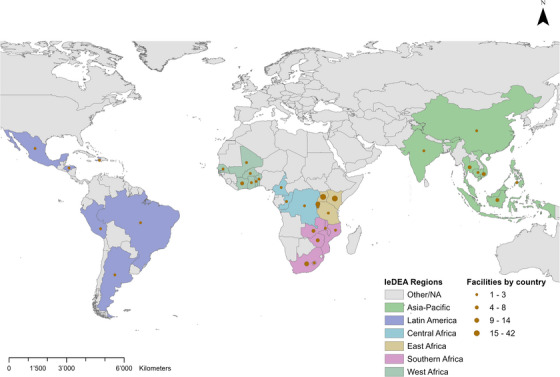
Surveyed HIV care facilities, by country and by the International epidemiology Databases to Evaluate AIDS region (*n* = 175).

Of the 179 facilities in LMICs, four (2.2%) did not complete the survey (Figure 1). Thus, we included 175 facilities in the study from six IeDEA regions: Asia‐Pacific (*n* = 30), Latin America (*n* = 8), Central Africa (*n* = 21), East Africa (*n* = 74), Southern Africa (*n* = 28) and West Africa (*n* = 14) (Figure 2 and Table S1). Out of the 175 facilities, 48% (84/175) were in lower‐middle‐income countries, 30% (52/175) in low‐income countries and 22% (39/175) in upper‐middle‐income countries (Table 1). Almost half of the facilities (83/175, 47%) were in countries with medium HIV prevalence, 33% (57/175) in countries with high HIV prevalence and 20% (35/175) in countries with low HIV prevalence.

**Table 1:** Characteristics of included health facilities by the International epidemiology Databases to Evaluate AIDS region

		Overall	Asia‐Pacific	Latin America	Central Africa	East Africa	Southern Africa	West Africa
Characteristic	Subgroups	*N* = 175 (100%) *N* (%)	*n* = 30 (17.1%) *n* (%)	*n* = 8 (4.6%) *n* (%)	*n* = 21 (12%) *n* (%)	*n* = 74 (42.3%) *n* (%)	*n* = 28 (16%) *n* (%)	*n* = 14 (8%) *n* (%)
Country income designation^a^	Low income	52 (30.0)	0 (0.0)	1 (12.5)	16 (76.2)	29 (39.2)	3 (10.7)	3 (21.4)
	Lower‐middle income	84 (48.0)	11 (36.7)	1 (12.5)	5 (23.8)	45 (60.8)	11 (39.3)	11 (78.6)
	Upper‐middle income	39 (22.0)	19 (63.3)	6 (75)	0 (0.0)	0 (0.0)	14 (50.0)	0 (0.0)
HIV prevalence^a^	Low (< 1%)	35 (20.0)	22 (73.3)	7 (87.5)	1 (4.8)	0 (0.0)	0 (0.0)	5 (35.7)
	Medium (1−4.9%)	83 (47.0)	8 (26.7)	1 (12.5)	20 (95.2)	45 (60.8)	0 (0.0)	9 (64.3)
	High (≥ 5%)	57 (33.0)	0 (0.0)	0 (0.0)	0 (0.0)	29 (39.2)	28 (100.0)	0 (0.0)
Facility type	Health centre	88 (50.2)	4 (13.3)	0 (0.0)	12 (57.1)	54 (73.0)	15 (53.6)	3 (21.4)
	District hospital	15 (8.6)	0 (0.0)	0 (0.0)	0 (0.0)	12 (16.2)	1 (3.6)	2 (14.3)
	Regional, provincial or university hospital	64 (36.6)	26 (86.7)	8 (100.0)	9 (42.9)	7 (9.5)	5 (17.8)	9 (64.3)
	Unknown	8 (4.6)	0 (0.0)	0 (0.0)	0 (0.0)	1 (1.3)	7 (25.0)	0 (0.0)
Residence of the population served at the facilities	Predominantly urban	49 (28.0)	10 (33.3)	8 (100.0)	7 (33.3)	1 (1.4)	16 (57.1)	7 (50.0)
	Predominantly rural	42 (24.0)	0 (0.0)	0 (0.0)	2 (9.5)	37 (50.0)	3 (10.7)	0 (0.0)
	Mixed urban/rural	84 (48.0)	20 (66.7)	0 (0.0)	12 (57.1)	36 (48.6)	9 (32.1)	7 (50.0)
Types of clients served at the HIV facilities^b^	Children (0−9 years of age)	157 (89.7)	20 (66.7)	6 (75.0)	21 (100.0)	71 (95.9)	27 (96.4)	12 (85.7)
	Adolescents/young adults (10−24 years of age)	165 (94.3)	25 (83.3)	7 (87.5)	21 (100.0)	71 (95.9)	28 (100.0)	13 (92.9)
	Adults—general population (≥ 20 years of age)	152 (86.9)	19 (63.3)	7 (87.5)	21 (100.0)	73 (98.6)	25 (89.3)	7 (50.0)
	All age groups	134 (76.6)	10 (33.3)	5 (62.5)	21 (100.0)	69 (93.2)	24 (85.7)	5 (35.7)

^a^ Based on publicly available information by country.

^b^ Each subgroup was captured as a binary variable and answer yes is presented. All other variables are mutually exclusive and sum up to 100%.

### 3.1 Facility characteristics

Half of the facilities (88/175, 50%) were health centres; 37% (64/175) regional, provincial or university hospitals; and 9% (15/175) district hospitals (Table 1). Overall, 84/175 (48%) facilities served populations living in mixed urban/rural areas, 49/175 (28%) predominantly urban and 42/175 (24%) predominantly rural, with variations across regions. Most facilities (157/175, 90%) provided HIV services to children, 94% (165/175) to adolescents and 87% (152/175) to adults. Overall, 77% (134/175) provided HIV services to all age groups.

### 3.2 DSD for HIV care

Three‐quarters of facilities (133/175, 76%) reported providing DSD for HIV care in general, with the Asia‐Pacific (8/30, 27%) and Latin America (3/8, 37%) regions having the lowest proportion, and East Africa having the highest proportion of facilities offering DSD (69/74, 93%) (Table 2). While 91% of facilities (121/133) reported providing DSD for HIV treatment, only 61% (81/133) provided DSD for HIV testing and 59% (79/133) provided DSD for ART initiation. Almost half of the facilities (64/133, 48%) reported providing DSD for all three services. None of the three facilities in Latin America reported providing DSD for HIV treatment.

**Table 2:** Differentiated service delivery (DSD) implementation at healthcare facilities for HIV clients, by the International epidemiology Databases to Evaluate AIDS region

		Overall	Asia‐Pacific	Latin America	Central Africa	East Africa	Southern Africa	West Africa
Characteristic	Subgroups	*N* = 175 (100%) *N* (%)	*n* = 30 (17.1%) *n* (%)	*n* = 8 (4.6%) *n* (%)	*n* = 21 (12%) *n* (%)	*n* = 74 (42.3%) *n* (%)	*n* = 28 (16%) *n* (%)	*n* = 14 (8%) *n* (%)
Provision of DSD^a^	Yes	133 (76.0)	8 (26.7)	3 (37.5)	17 (81.0)	69 (93.2)	25 (89.3)	11 (78.6)
	No	42 (24.0)	22 (73.3)	5 (62.5)	4 (19.0)	5 (6.8)	3 (10.7)	3 (21.4)
Type of DSD provided^b^	HIV testing	81/133 (60.9)	3/8 (37.5)	3/3 (100.0)	9/17 (52.9)	45/69 (65.2)	15/25 (60.0)	6/11 (54.5)
	ART initiation	79/133 (59.4)	4/8 (50.0)	1/3 (33.3)	8/17 (47.1)	42/69 (60.9)	17/25 (68.0)	7/11 (63.6)
	HIV treatment	121/133 (91)	6/8 (75.0)	0/3 (0.0)	17/17 (100.0)	65/69 (94.2)	23/25 (92.0)	10/11 (90.9)
	All three services	64/133 (48.1)	1/8 (12.5)	0/3 (0.0)	8/17 (47.1)	36/69 (52.2)	14/25 (56.0)	5/11 (45.4)
Standard frequency of refills for clients who are stable on ART/MMD^a^	Monthly	24 (13.7)	10 (33.3)	3 (37.5)	0 (0.0)	6 (8.1)	4 (14.3)	1 (7.1)
	Every 3 months	122 (69.7)	15 (50.0)	3 (37.5)	18 (85.7)	65 (87.8)	13 (46.4)	8 (57.1)
	Every 6 months	17 (9.7)	4 (13.3)	1 (12.5)	2 (9.5)	2 (2.7)	5 (17.9)	3 (21.4)
	Other	12 (6.9)	1 (3.3)	1 (12.5)	1 (4.8)	1 (1.4)	6 (21.4)	2 (14.3)
DSD for HIV treatment models offered								
Clients served via DSD for HIV treatment^c^	Clients presenting/returning to care with advanced HIV disease^d^	61/121 (50.4)	4/6 (66.7)	0 (0.0)	3/17 (17.6)	38/65 (58.5)	12/23 (52.2)	4/10 (40.0)
	Clients presenting/returning to care when clinically well	80/121 (66.1)	1/6 (16.7)	0 (0.0)	7/17 (41.2)	50/65 (76.9)	16/23 (69.6)	6/10 (60.0)
	Clients clinically stable on ART	110/121 (90.9)	2/6 (33.3)	0 (0.0)	15/17 (88.2)	61/65 (93.8)	23/23 (100.0)	9/10 (90.0)
	Clients on ART with virologic/therapeutic failure^d^	69/121 (57.0)	4/6 (66.7)	0 (0.0)	5/17 (29.4)	38/65 (58.5)	15/23 (65.2)	7/10 (70.0)

Abbreviations: ART, antiretroviral therapy; DSD, differentiated service delivery; MMD, multi‐month dispensing.

^a^ Mutually exclusive variables sum up to 100%. All other variables are binary and answer yes is presented.

^b^ Denominator based only on facilities providing DSD.

^c^ Denominator based only on facilities providing any DSD for HIV treatment models.

^d^ Definitions: Advanced HIV disease: CD4 < 200 cells/mm^3^ and/or WHO Clinical Stage 4 disease. Virologic failure: “unstable clients” on ART > 1 year.

### 3.3 MMD and ART refill frequency

The most common frequency of ART refills for clinically stable clients across all regions was 3 months (122/175, 70%), followed by monthly (24/175, 14%), and every 6 months (17/175, 10%) (Table 2). The Central African (18/21, 86%) and Eastern African (65/74, 88%) regions reported higher proportions of 3MMD, while monthly refills were highest for the Asia‐Pacific (10/30, 33%) and Latin American (3/8, 37%) regions.

### 3.4 Factors associated with DSD availability

**Figure 3:**
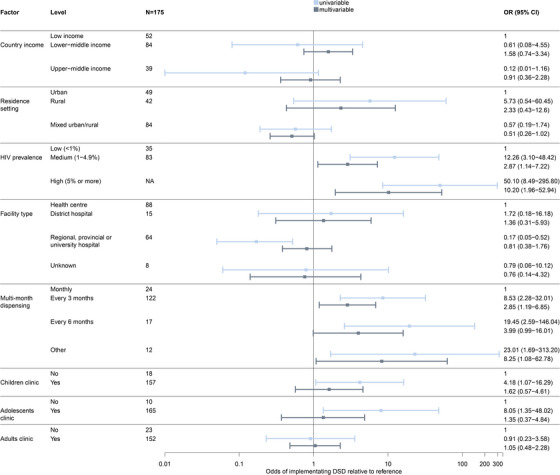
Univariable and multivariable model exploring factors associated with differentiated service delivery implementation. The adjusted model accounts for all factors listed in the figure.

In multivariable models, facilities located in countries with a medium HIV prevalence were twice as likely (aOR 2.87; 95% CI 1.14−7.22) and those with a high HIV prevalence were 10 times as likely (aOR 10.20; 95% CI 1.96−52.94) to have implemented DSD than those with a low HIV prevalence. Similarly, facilities offering 3MMD were twice as likely (aOR 2.85; 95% CI 1.19−6.85) to implement DSD than those on a monthly schedule (Figure 3).

### 3.5 DSD for HIV treatment models

DSD for HIV treatment was used for clinically stable clients on ART in almost all facilities (110/121, 91%), for clients presenting or returning to care when clinically well (80/121, 66%) and for clients on ART with virological failure in more than half of them (69/121, 57%) (Table 2).

**Figure 4:**
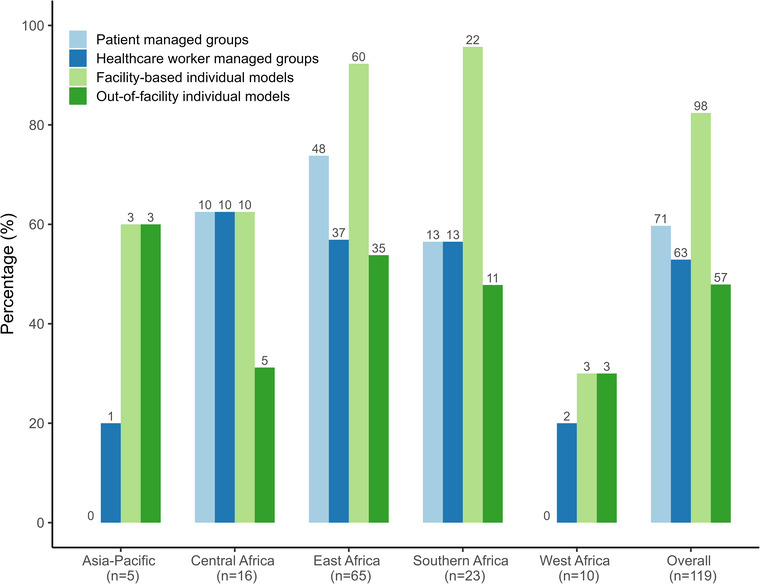
Availability of differentiated service delivery for HIV treatment models by the International epidemiology Databases to Evaluate AIDS region in 2019. *None of the facilities in Latin America reported providing differentiated service delivery for HIV treatment.

Of the 119/121 facilities providing information on DSD for HIV treatment models, 98/119 (82%) reported offering facility‐based individual models, 71/119 (60%) patient‐managed group models, 63/119 (53%) healthcare worker‐managed group models and 57/119 (48%) out‐of‐facility individual models (Figure 4). These models were not mutually exclusive, and facilities could implement one or more models concurrently. Facility‐based individual models were particularly common among facilities in East (60/65, 92%) and Southern Africa (22/23, 96%). Almost half of the facilities (47/119, 40%) reported the introduction of at least one of the four DSD for HIV treatment models in 2018 (Supporting information Table S2 and Figure S1).

### 3.6 Dedicated HIV clinics for specific population groups

Most facilities reported offering dedicated HIV clinics for pregnant or breastfeeding women (150/175, 86%) and clients with comorbidities or opportunistic infections (142/175, 81%) (Table 3). Overall, fewer facilities reported offering dedicated HIV clinics for key population groups with high HIV acquisition risk. Less than half of the facilities offered dedicated clinics for gay men and other men who have sex with men (57/175, 33%) or female sex workers (71/175, 41%), and less than a third offered dedicated clinics for transgender individuals (46/175, 26%), people who inject drugs (46/175, 26%) or incarcerated populations (47/175, 27%), with variations across regions.

**Table 3:** Availability of dedicated HIV clinics providing care to various population groups, by the International epidemiology Databases to Evaluate AIDS region

Population group	Overall *N* = 175 (100%) *N* (%)	Asia‐Pacific *n* = 30 (17.1%) *n* (%)	Latin America *n* = 8 (4.6%) *n* (%)	Central Africa *n* = 21 (12%) *n* (%)	East Africa *n* = 74 (42.3%) *n* (%)	Southern Africa *n* = 28 (16%) *n* (%)	West Africa *n* = 14 (8%) *n* (%)
Pregnant/breastfeeding women	150 (85.7)	22 (73.3)	6 (75.0)	21 (100.0)	71 (95.9)	22 (78.6)	8 (57.1)
Family care clinics	86 (49.1)	15 (50.0)	2 (25.0)	16 (76.2)	30 (40.5)	18 (64.3)	5 (35.7)
Men	113 (64.6)	19 (63.3)	6 (75.0)	19 (90.5)	42 (56.8)	20 (71.4)	7 (50.0)
Clients with comorbidities or opportunistic infections	142 (81.1)	28 (93.3)	6 (75.0)	20 (95.2)	52 (70.3)	23 (82.1)	13 (92.9)
Female sex workers	71 (40.6)	16 (53.3)	5 (62.5)	18 (85.7)	21 (28.4)	9 (32.1)	2 (14.3)
Gay men and other men who have sex with men	57 (32.6)	20 (66.7)	7 (87.5)	9 (42.9)	13 (17.6)	6 (21.4)	2 (14.3)
Transgender individuals	46 (26.3)	18 (60.0)	6 (75.0)	5 (23.8)	10 (13.5)	6 (21.4)	1 (7.1)
People with substance use disorders	55 (31.4)	17 (56.7)	5 (62.5)	4 (19.0)	23 (31.1)	6 (21.4)	0 (0.0)
People who inject drugs	46 (26.3)	17 (56.7)	3 (37.5)	3 (14.3)	17 (23.0)	5 (17.9)	1 (7.1)
People with mental health disorders	85 (48.6)	21 (70.0)	4 (50.0)	14 (66.7)	28 (37.8)	16 (57.1)	2 (14.3)
Mobile populations	75 (42.9)	12 (40.0)	2 (25.0)	12 (57.1)	32 (43.2)	14 (50.0)	3 (21.4)
Incarcerated populations/prisoners	47 (26.9)	8 (26.7)	3 (37.5)	6 (28.6)	18 (24.3)	8 (28.6)	4 (28.6)
People living with disabilities	101 (57.7)	22 (73.3)	5 (62.5)	19 (90.5)	33 (44.6)	14 (50.0)	8 (57.1)

## 4 DISCUSSION

We analysed site‐survey data from 175 HIV treatment and care facilities in 35 LMICs to assess the global implementation of DSD before the COVID‐19 pandemic. Three‐quarters of facilities reported providing DSD services in HIV care, with DSD more common for HIV treatment than for HIV testing or ART initiation, with variations across regions. While nearly 90% of facilities in Africa reported offering DSD services, outside of Africa it was less than a third, with none of the surveyed facilities in Latin America offering DSD for HIV treatment. Less than a tenth of facilities reported using 6MMD for clinically stable clients, with most facilities offering 3MMD. Less than half of the facilities reported dedicated care for key population groups, such as female sex workers or people who inject drugs.

In our study, facility‐based models were more frequently implemented than community‐based models. While this result is consistent with previous studies reporting client's preferences for facility‐based HIV services [19, 35, 36, 37, 38, 39, 40], it is important to consider that such preferences may reflect what participants know and what is most accessible to them. In settings where facility‐based individual models combine clinical visits with extended ART refills, this approach can reduce the time spent at the facility [41]. Combined with fast‐track facility‐based drug refills, MMD can provide a cost‐ and time‐efficient way of accessing ART. In a study in Zambia, clinically stable HIV clients in urban settings preferred facility‐based individual models over group or community‐based ones because they offered extended MMD and fast‐track visits [42]. However, implementing facility‐based models can be challenged by limited resources, including dedicated personnel or space, particularly at smaller facilities [8, 9, 39]. In contrast, people living in rural areas of Zambia preferred community‐based models, which saved them travel‐associated time and costs [42]. Of note, clients of community‐based HIV services were shown to remain stable on care and maintain good adherence to treatment, without an increased risk of loss to follow‐up than those in facility‐based models [43].

Our finding that 3MMD was the most frequent ART refill interval, with 6MMD rarely available, is consistent with other studies conducted in LMICs showing that most ART programmes provided 2 or 3 months of ART refills between 2016 and 2019 [36, 44]. The advantages of longer ART refills for clients include time and cost savings, minimised work interruptions, and potentially diminished exposure to HIV‐related stigma and discrimination, as clinic visits decrease [45, 46]. Studies comparing MMD strategies showed similar retention in care for 6MMD and 3MMD [47, 48, 49]. On the healthcare provision side, longer ART refill schedules can contribute to decongesting facilities and decreasing providers’ workloads [45, 46]. One of the main obstacles to the implementation of 6MMD lies with the frequent drug stock‐outs, especially in LMICs [9, 37].

We observed an association between higher HIV prevalence at country level and the implementation of DSD. HIV prevalence emerges as a determinant influencing the relevance and acceptability of DSD models within healthcare systems [50]. Regions facing a significant burden of diseases like HIV can exhibit a better implementation of DSD to better suit the various people's need, and thus to improve retention in care and clinical outcomes [51, 52]. Although in our model country income was not associated with DSD implementation, other studies have noted that upper‐ and lower‐middle‐income countries had rapidly implemented “treat all” and provided universal HIV treatment to PWH [53, 54, 55, 56], accompanied by many interventions to accelerate treatment uptake. Moreover, other factors can influence the implementation of DSD such as national policies, research programmes and funding sources. Others have shown that facilities supported by external funding can embrace more DSD initiatives [37, 55, 57, 58]. Future research should further examine how economic and political dynamics influence the implementation and long‐term sustainability of DSD.

In our study, few facilities offered dedicated clinics for key population groups. Key populations often suffer from limited access to a wide range of HIV services, especially if not targeted to their specific needs [2]. Consequently, key population groups are often at increased HIV acquisition risk and experience higher HIV prevalence than the general population, with lower access to ART [59, 60]. Discrimination and social exclusion can hinder access to HIV services for key population groups in countries where these populations are criminalised [61]. This highlights the need for dedicated services tailored to these groups, including prevention, testing, ART delivery and long‐term prevention [61, 62]. Key population groups may be more likely to access HIV services in trusted environments, with tailored DSD models specifically addressing stigma and discrimination which are prominent concerns in these populations [5, 63, 64].

To our knowledge, this is one of few global analyses examining the implementation of DSD in LMICs [65]. The main strength of our study is that it captured the scale of DSD across a large sample of HIV care facilities in LMICs globally, using standardised questionnaires. However, our data do not provide information on the actual client‐level uptake of DSD, its acceptability, nor its sustainability, which are essential elements to understand the success of implementation processes but were beyond the scope of this site‐level survey. Furthermore, we acknowledge that while being a global consortium, IeDEA is mainly comprised of large sites and programmes, including referral and university teaching hospitals that serve large numbers of clients and provide advanced or specialised HIV care. Facilities participating in IeDEA are likely not representative of all facilities that provide HIV care within LMICs generally. Our survey results may overestimate the implementation of DSD in sub‐Saharan Africa settings if rollout treatment guidelines lag at peripheral, lower‐level facilities or among clinics serving key populations. Additionally, IeDEA comprises only eight facilities in Latin America, which are selected based on the availability of electronic data. Another limitation of our survey is that it is self‐reported, and thus subject to recall bias and social desirability bias. Moreover, our data may not fully reflect the current context due to evolving policies and healthcare practices. Our analysis focused on service delivery practices in 2019, 1 year prior to the COVID‐19 pandemic, yet many sites accelerated the implementation of DSD strategies in response to the pandemic to minimise patient visits and maintain client retention in care [51, 66–68]. Moreover, our study did not explore novel DSD strategies implemented during the COVID‐19 pandemic to maintain client retention in care while minimising visits to healthcare facilities [22, 23, 69]. For example, in countries supported by the United States President's Emergency Plan for AIDS Relief (PEPFAR), MMD increased access to ART during the COVID‐19 pandemic [70]. Nevertheless, despite the age of our data, they are still relevant to the current push towards client‐centred HIV care and provide a timely global overview of efforts being made towards DSD implementation across different settings. We acknowledge that newer developments may have influenced DSD implementation and should be considered when interpreting our findings. We also believe that collecting more data on specific DSD components is essential to better understand the nuanced impact of national policies across countries. At present, we are not using scales to assess DSD in the post‐COVID era, and while comparisons with pre‐COVID data may offer valuable insights, such analyses are not planned within the scope of this work. Finally, we recognise that our model exploring factors associated with DSD implementation combined data collected at facility and country levels, which may not reflect the exact same setting.

## 5 CONCLUSIONS

Most of the HIV care facilities in LMICs had implemented a range of DSD for HIV treatment models by 2019. The majority offered facility‐based models and 3MMD for clinically stable clients. Fewer facilities used community‐based models and extended MMD. Efforts to expand DSD into HIV testing and ART initiation and offer extended MMD in resource‐constrained settings would contribute to further alleviate the burden on healthcare services and PWH, while improving long‐term adherence and retention in care.

## COMPETING INTERESTS

The authors have declared no competing interests.

## AUTHORS’ CONTRIBUTIONS

International epidemiology Databases to Evaluate AIDS performed the site‐level survey. VK, CT, MS, BC, JH, TT, CWW, SMF, GM, YC‐V, REL, IR, DMZ, KP, DMN, JP, KW‐K, CBM, OET, SK, RAA, SMA, GF and KM provided survey data. MB designed the research study. NVFV, FH and EZ analysed the data. NVFV, EZ and MB wrote the first draft. CWW assisted with survey design. All authors have read and approved the final manuscript.

## FUNDING

The International Epidemiology Databases to Evaluate AIDS (IeDEA) is supported by the U.S. National Institutes of Health's National Institute of Allergy and Infectious Diseases, the *Eunice Kennedy Shriver* National Institute of Child Health and Human Development, the National Cancer Institute, the National Institute of Mental Health, the National Institute on Drug Abuse, the National Heart, Lung, and Blood Institute, the National Institute on Alcohol Abuse and Alcoholism, the National Institute of Diabetes and Digestive and Kidney Diseases, and the Fogarty International Center: Asia‐Pacific, U01AI069907; CCASAnet, U01AI069923; Central Africa, U01AI096299; East Africa, U01AI069911; NA‐ACCORD, U01AI069918; Southern Africa, U01AI069924; West Africa, U01AI069919. Informatics resources are supported by the Harmonist project, R24AI24872. Data management resources were supported by the Research Electronic Data Capture (REDCap) grant UL1 TR000445 from NCATS/NIH.

## DISCLAIMER

This work is solely the responsibility of the authors and does not necessarily represent the official views of any of the institutions mentioned above.

## Supporting information




**Table S1**: List of countries per region and number of facilities per country.
**Table S2**: Year of implementation of DSD for HIV treatment models.
**Figure S1**: Year of introduction of DSD for HIV treatment models by IeDEA region.
**Text S1**: Acknowledgements.
**Text S2**: IeDEA 2020 Site Assessment Survey.

## Data Availability

People interested in accessing the IeDEA consortium data for research purposes may contact the corresponding author for more information or see our website at https://www.iedea.org/.
